# Critical roles of rare species in the anaerobic ammonium oxidizing bacterial community in coastal sediments

**DOI:** 10.1007/s42995-025-00315-8

**Published:** 2025-07-17

**Authors:** Yu Zhang, Mingming Chen, Rui Du, Ehui Tan, Shuh-Ji Kao, Yao Zhang

**Affiliations:** 1 State Key Laboratory of Marine Environmental Science and College of Ocean and Earth Sciences, Xiamen University, Xiamen, 361005 China; 2 State Key Laboratory of Marine Resource Utilization in South China Sea, School of Marine and Engineering, Hainan University, Haikou, 570228 China

**Keywords:** Anammox bacteria, Community structure, Community assembly, Co-occurrence network, Rare species

## Abstract

Anaerobic ammonium oxidation (anammox) plays a critical role in nitrogen loss in estuarine and marine environments. However, the mechanisms underlying the formation and maintenance of the anammox bacterial community remain unclear. This study analyzed the anammox bacterial diversity, community structure, and interspecific relationships in three estuaries along the Chinese coastline —the Changjiang Estuary (CJE), the Oujiang Estuary (OJE), and the Jiulong River Estuary (JLE) — as well as the South China Sea (SCS) to elucidate their community assembly mechanisms. The results indicated that the anammox bacterial community exhibited the highest ammonium concentration as well as the Shannon’s diversity index reflecting both species richness and evenness in the JLE. The lowest Shannon index was observed in the SCS. However, the anammox bacterial species richness was greatest in the CJE. *Candidatus* Scalindua was the predominant anammox bacteria identified in the coastal sediments, especially in the SCS sediments. *Candidatus* Brocadia and *Candidatus* Kuenenia were more abundant in the estuarine sediments, particularly in JLE, than in the SCS. Phylogenetic analysis revealed distinct differentiation among *Candidatus* Scalindua, *Candidatus* Brocadia, and *Candidatus* Kuenenia, with the former exhibiting a greater level of diversity. There was significant spatial heterogeneity in the anammox bacteria across the four regions, characterized by distinct distribution patterns for rare species. Low-abundance (rare) bacteria thrived in their native habitats, whereas abundant taxa displayed greater dispersal capabilities. An analysis of the community assembly mechanism suggested that ecological drift predominantly shaped the overall anammox bacterial community in the coastal sediments. Rare species were more susceptible to dispersal limitations and environmental selection. Co-occurrence network analysis identified *Candidatus* Scalindua as a keystone genus and highlighted that rare species may play a crucial role in maintaining the ecological stability of the anammox bacterial community in coastal sediments.

**Supplementary Information:**

The online version contains supplementary material available at 10.1007/s42995-025-00315-8.

## Introduction

Nitrogen (N) is an essential element for microorganisms and is a limiting nutrient for primary productivity in oceans (Devol 2015; Stein and Klotz 2016). Significant amounts of reactive N produced by human activities have entered the oceans via river inputs and atmospheric deposition, doubling the bioavailable N content in estuaries and coastal areas since the last century. This influx has led to numerous harmful ecological and environmental effects, including eutrophication, hypoxia from decomposition, and acidification (Cai et al. 2011; Damashek and Francis 2017; Francis et al. 2007; Galloway et al. 1995; Rabalais et al. 2009). Consequently, these changes accelerate N loss through denitrification and anaerobic ammonium oxidation (anammox) pathways in coastal regions, particularly in benthic sediments (Li et al. 2021). Estuaries and coastal areas are major regions of N loss within the marine systems (Dong et al. 2009).

For long, denitrification, which produces dinitrogen (N_2_) and nitrous oxide (N_2_O), was considered the only pathway for removing reactive N from various environments (Francis et al. 2007). However, the discovery of the anammox process in the 1990 s, which anaerobically converts ammonium (NH_4_^+^) and nitrite (NO_2_^–^) to N_2_ without producing N_2_O, remarkably expanded our understanding of the N cycle (Kartal et al. 2007a; Mulder et al. 1995; van de Graaf et al. 1995). Through molecular biology and isotope pairing techniques, numerous measurements of anammox bacteria and their potential activities indicate that anammox could account for a substantial fraction of benthic N_2_ production in many coastal regions (Brin et al. 2014; Crowe et al. 2012; Dale et al. 2009; Hou et al. 2013; Trimmer et al. 2003; Wang et al. 2012). The diversity and community structure of the anammox bacteria influence the anammox flux. The metacommunity (i.e., community compositions across sampling sites) and the potential activity of anammox bacteria have been widely studied in various marine and coastal ecosystems. However, the mechanisms by which their diversity is shaped and maintained—specifically, those of community assembly in benthic sediments—remain largely unexplored. Although the ecological importance of rare microbial taxa has gained recent substantial recognition, their potential roles in shaping the network dynamics and community assembly processes of anammox bacteria remain poorly understood.

Based on high-throughput sequencing of their 16S rRNA gene, this study analyzed the diversity, community structure, and community assembly of anammox bacteria in estuaries and coastal areas affected by human activities, including the Changjiang Estuary (CJE), Oujiang Estuary (OJE), and Jiulong River Estuary (JLE), as well as the northeastern South China Sea (SCS). In particular, this study is the first to focus on the role of the rare anammox bacterial taxa in ecosystems to elucidate the mechanisms underlying the anammox bacterial community assembly and co-occurrence network patterns, enhancing our understanding of the functional roles of these microorganisms within ecological networks.

## Materials and methods

### Sample collection

**Fig. 1:**
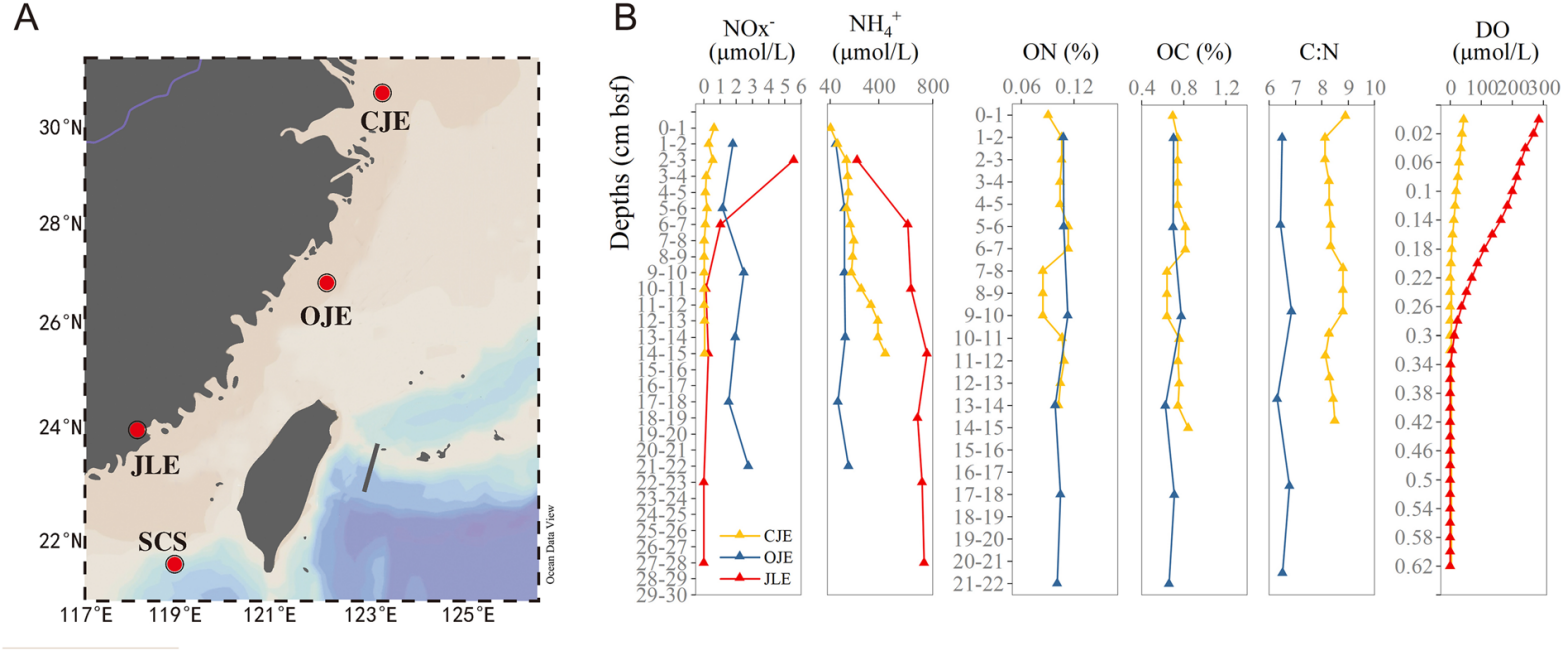
**A** Sampling stations and **B** environmental parameters. *CJE* Changjiang Estuary, *OJE* Oujiang Estuary, *JLE* Jiulong River Estuary, *SCS* South China Sea, NO_x_^–^ nitrite + nitrate (only nitrate in the JLE), NH_4_^+^ ammonium, *ON* organic nitrogen, *OC* organic carbon, *C:N* ratios of organic matter, *DO* dissolved oxygen. The map of the sampling regions was generated using the Ocean Data View software (https://odv.awi.de/)

A sediment core from the JLE (30 cm in depth) was sampled during a cruise aboard *Ocean II* in December 2015. Sediment cores from the CJE (15 cm in depth) and OJE (22 cm in depth) were collected in July 2017 and August 2021, respectively, aboard *Yanping II.* A sediment core from the SCS (34 cm in depth) was collected in May 2018 aboard *Tan Kah Kee*. The JLE and OJE cores were subsampled at 4 cm intervals, yielding seven and six subsamples, respectively. The CJE and SCS cores were subsampled at high-resolution intervals of 1–2 cm, resulting in 15 and 17 subsamples, respectively. Detailed information about the sampling sites is provided in Fig. 1A and Table S1.

### Chemical parameter analysis

The sediments were dried and acidified by adding 1 N HCl to remove the inorganic carbon. The organic C and N contents of the sediment were measured using a Carlo-Erba EA 2100 elemental analyzer, Thermo Scientific, MA, USA (Kao et al. 2008). The nitrate (NO_3_^–^), nitrite (NO_2_^–^), and NH_4_^+^ concentrations in the sediment pore water were ascertained using an AA3 nutrient auto-analyzer (Bran + Luebbe GmbH, Germany). The dissolved oxygen (DO) levels were measured utilizing an OX 50 oxygen microsensor (Unisense AS, Aarhus N, Denmark) with a high spatial resolution interval of 0.2 mm (Tan et al. 2019). Before measuring the DO, a two-point calibration with saturations of 0% and 100% was performed.

### PCR and High-throughput sequencing

Total DNA from 0.5 g of each of the wet sediment samples was extracted utilizing the FastDNA™ SPIN Kit for soil DNA extraction (MP Biomedical, CA, USA), following the manufacturer’s instructions. The DNA was quantified and assessed for purity employing a Qubit fluorometer (Thermo Scientific, USA) and a NanoDrop 2000 spectrophotometer (Thermo Scientific, USA). The DNA samples were stored at  – 20 ℃. The anammox bacterial 16S rRNA gene was amplified with the primers Brod541F and Amx820R (Penton et al. 2006; Schmid et al. 2000). Each PCR reaction mix had a total volume of 50 μL, containing 25 μL of Premix Ex Taq (Takara Bio Inc., Shiga, Japan), 2 μL each of the forward and reverse primers (20 μmol/L), 2 μL of the DNA template, 1 μL of bovine serum albumin (Takara Bio), and 18 μL of RNase-free Water (Takara Bio). The thermal program for PCR comprised an initial 5 min at 95 ℃; followed by 35 cycles of 45 s at 95 ℃, 30 s at 56 ℃, and 50 s at 72 ℃; with a final extension step of 10 min at 72 ℃ (Han et al. 2013; Li et al. 2010). The amplicons were separated using 1% agarose gel electrophoresis at 125 V for 25 min and were subjected to high-throughput sequencing at Majorbio Biotech, China.

### Sequence diversity analysis

A total of 1,019,646 raw sequences were obtained. They were denoised using the Sickle program Version 1.33 (https://bioweb.pasteur.fr/packages/pack@sickle@1.33) and then assigned to operational taxonomic units (OTUs) with 98% similarity. Chimeras were removed utilizing the QIIME 2 program (QIIME 2 Core 2021; https://qiime2.org/). The OTUs were then aligned and annotated, employing a specific gene database for anammox bacteria (https://data.mendeley.com/datasets/27vckf6mgk/3) (Wen et al. 2020). The alpha diversity indices (Shannon–Wiener diversity and Abundance-based Coverage Estimator (ACE) richness) of the anammox bacteria were calculated utilizing the QIIME 2 package. The nearest taxon index (NTI) was calculated employing *R* version 3.6.3 (https://cran.r-project.org/bin/windows/base/). The Bray–Curtis dissimilarity and Euclidean distance were analyzed using the ecology-applicable PRIMER-e software (https://www.primer-e.com/software). The UniFrac dissimilarity of the anammox bacterial communities was assessed using *R* packages. One-way ANOVA and Tukey’s LSD tests were conducted employing GraphPad Prism 10.1 (https://www.graphpad.com/). The spatial heterogeneity of the anammox bacteria was analyzed utilizing nonmetric multidimensional scaling (NMDS) and multivariate nonparametric tests (ANOSIM [analysis of similarities]) in PRIMER-e.

Based on their relative abundances, microbial communities were classified into the following categories (Dai et al. 2017): rare taxa (RT), abundance less than 0.1% in all samples; abundant taxa (AT), abundance greater than 1% in all samples; moderate taxa (MT), abundance between 0.1% and 1% in all samples; conditionally rare taxa (CRT), abundance less than 1% in all samples and less than 0.1% in some samples; conditionally abundant taxa (CAT), abundance greater than 0.1% in all samples and greater than 1% in some samples; finally, conditionally rare or abundant taxa (CRAT), abundance less than 0.1% in some samples and greater than 1% in others.

### Construction of the phylogenetic tree

The OTU sequences with relative abundances greater than 0.01%, along with the reference and outgroup sequences, were aligned using the Neighbor-Joining algorithm. Before constructing the phylogenetic trees, the MEGA 7.0 software (https://www.megasoftware.net/) was used to determine the most suitable model. Multiple sequence alignment employed the MAFFT 7.520 program (https://mafft.cbrc.jp/alignment/software/). Phylogenetic tree construction and visualization were performed utilizing the MEGA 7.0 and FigTree v1.4.4 (https://tree.bio.ed.ac.uk/software/figtree/) software, respectively.

### Quantification of the community assembly processes

The beta mean nearest taxon distance (βMNTD) matrix and the Raup-Crick index based on the Bray–Curtis dissimilarity (RC_bray_) matrix were calculated by applying the null model using the “picante” or “iCAMP” packages in *R*. The ecological processes quantified were: Heterogeneous Selection (HeS) (βNTI > 1.96), Homogeneous Selection (HoS) (βNTI <  – 1.96), Dispersal Limitation (DL) (– 1.96 < βNTI < 1.96 and RC_bray_ > 0.95), Homogenizing Dispersal (HD) (– 1.96 < βNTI < 1.96 and RC_bray_ <   –  0.95), and Drift (DR) (– 1.96 < βNTI < 1.96 and  – 0.95 < RC_bray_ < 0.95) (Stegen et al. 2013, 2012). Quantifying ecological processes via the null model necessitates a significant phylogenetic signal under the optimal niche conditions of the OTUs (Stegen et al. 2013; Zhou and Ning 2017), which was detected using the *R* package “microeco.” Marked correlations between phylogenetic distances and niche differences indicate the presence of phylogenetic signals. Significant phylogenetic signals were observed at relatively close phylogenetic distances (Fig. S1).

### Topological analysis of the co-occurrence networks

The topological parameters of the network co-occurrence model were analyzed utilizing the “sparcc” and “igraph” packages of *R* and Gephi 0.9 (https://gephi.org/users/download/). The pairwise Spearman’s correlations between OTUs were retained only if the correlation coefficient was greater than 0.6 and the *P*-value was less than 0.05, with adjustments made using the Benjamini–Hochberg method. The power–law distribution of the ecological network model (Fig. S2) verified the nonrandom nature of the co-occurrence network. Modularity analysis of the anammox bacterial ecological network was performed employing Gephi 0.9, identifying modules that comprised more than 10% of the total nodes as the main ones. Keystone species in the networks were identified by determining the microbial taxa with the most interactions. Specifically, key populations were identified utilizing a Zi-Pi plot, which assessed the roles of the nodes within the networks (intra-module connectivity: Zi; inter-module connectivity: Pi). Zi and Pi were calculated using the “igraph” package in *R* (Deng et al. 2012; Zheng et al. 2023). Keystone species were categorized into four types: (1) module hubs, which are nodes with high connectivity within different modules (Zi > 2.5 and Pi < 0.62); (2) connectors, which are nodes with high connectivity between the various modules (Zi < 2.5 and Pi > 0.62); (3) network hubs, nodes with high connectivity within the overall topological structure (Zi > 2.5 and Pi > 0.62); (4) peripherals, which are nodes with low intra- and inter-module connectivity (Zi < 2.5 and Pi < 0.62).

### Random forest and linear discriminant analyses

Random forest analysis (RFA) and linear discriminant analysis (LDA) analyzed the enrichment preference of the OTUs among different environments using the “randomForest” and “lefser” packages in *R*, respectively. The receiver operating characteristic curve derived from the RFA indicated that the model exhibited robust classification performance, demonstrating high prediction accuracy (Fig. S3).

## Results

### Environmental factors

Significant regional differences were observed in NH_4_^+^, NO_x_^–^ (NO_2_^–^ + NO_3_^–^), dissolved oxygen concentrations (DO), and C:N ratios of the organic matter across the estuarine sediments (Fig. 1B). The highest average NH_4_^+^ level was recorded in JLE (628.33 μmol/L), followed by CJE (220.48 μmol/L) and OJE (134.61 μmol/L), suggesting more severe eutrophication at JLE. The NH_4_^+^ levels in the subsurface sediments were relatively higher compared to those in the surface sediments. In contrast to NH_4_^+^ concentrations, NO_x_^–^ levels were relatively greater in the surface sediments compared to the subsurface sediments (Fig. 1B). The highest average NO_x_^–^ concentration was recorded in OJE (1.94 μmol/L), while CJE exhibited the lowest (0.17 μmol/L). The total organic carbon and nitrogen contents in OJE and CJE were ~ 0.8%, indicating no significant variation in the organic matter content (Fig. 1B). However, the average C:N ratio, an indicator of sediment activity and organic matter availability, was markedly higher in CJE (8.34) than in OJE (6.56) (ANOVA *P* < 0.05) (Fig. 1B). This result suggests that the organic matter at OJE is more available to heterotrophic microorganisms, while it is relatively refractory in CJE.

### Diversity of the anammox bacteria

The rarefaction curves plateaued with an increased number of reads, indicating that the sequencing depth was sufficient to capture a majority of the diversity (Fig. S4A and B). The coverage approached 0.99, demonstrating that nearly all OTUs in the community were detected (Fig. S4C). The sediment samples from the CJE, OJE, JLE, and SCS contained 2,114, 728, 930, and 1,412 OTUs, respectively, indicating greater richness at the CJE. Alpha diversity indices revealed that the ACE richness index in the CJE ranged from 879.30 to 1480.81, which was significantly higher than that in other ecosystems (ANOVA, *P* < 0.05). Notably, there was no remarkable difference in the ACE indices between the SCS and estuarine sediments, except for CJE (ANOVA, *P* > 0.05; Fig. S5A). JLE had the maximum Shannon index, reflecting both species richness and evenness at 1.84–2.77, remarkably higher than that in the OJE (1.31–1.66) and SCS (0.07–2.10) (ANOVA, *P* < 0.05). The CJE also had a relatively higher Shannon index, significantly exceeding that of SCS (ANOVA, *P* < 0.05) (Fig. S5B). The NTI index demonstrated a uniform pattern across the different marine sediments (ranging from 0.23 to 0.59), indicating no significant environmental filtering (Stegen et al. 2012) of anammox bacteria within coastal sediments (Fig. S5C).

### Phylogeny and community structure of the anammox bacteria

**Fig. 2:**
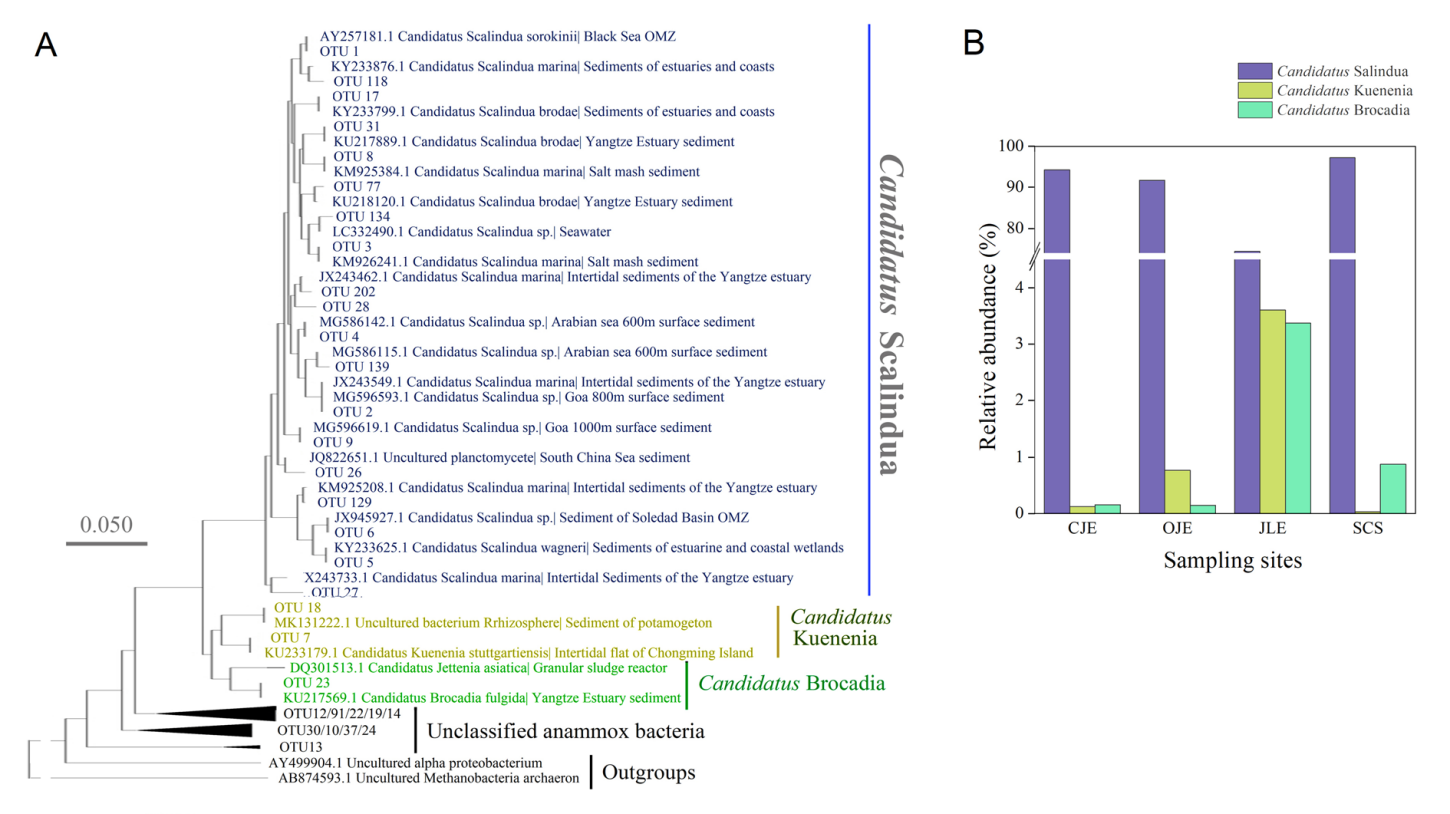
**A** Neighbor-Joining-based phylogenetic tree of anammox bacteria 16S rRNA gene, and **B** relative abundances of *Candidatus* Scalindua, *Candidatus* Kuenenia, and *Candidatus* Brocadia in the sediments. *CJE* Changjiang Estuary, *OJE* Oujiang Estuary, *JLE* Jiulong River Estuary, *SCS* South China Sea

Phylogenetics identified that *Candidatus* Scalindua, *Candidatus* Kuenenia, and *Candidatus* Brocadia formed three distinct clusters within the coastal sediments (Fig. 2A). The four OTUs with the highest relative abundances, OTU1, OTU2, OTU4 and OTU3, belonged to *Candidatus* Scalindua (Fig. 2A and Table S2), with averages of 32.57%, 16.79%, 13.29%, and 10.42%, respectively. These OTUs exhibited marked similarity to sequences retrieved from other marine and coastal sediment environments, with OTU1, OTU2, and OTU4 showing 99%–100% identity with sequences from the Black Sea, Goa, and the Arabian Sea, respectively. Furthermore, *Candidatus* Scalindua exhibited a diverse phylogenetic distribution, including *Candidatus* Scalindua marina, *Candidatus* Scalindua arabica, *Candidatus* Scalindua brodae, *Candidatus* Scalindua sorokinii, *Candidatus* Scalindua wagneri, and *Candidatus* Scalindua spp. Only three OTUs belonged to *Candidatus* Kuenenia and *Candidatus* Brocadia, with 98%–99% homology to sequences from the intertidal and estuarine sediments (Fig. 2A).

*Candidatus* Scalindua is the predominant anammox bacterium in the coastal sediments of China, with respective relative abundances of 97.2%, 94.2%, 91.8%, and 74.4% in the SCS, CJE, OJE, and JLE sediments (Fig. 2B). At the species level, *Candidatus* Scalindua marina and *Candidatus* Scalindua sorokinii were dominant in the CJE, OJE, and SCS sediments, whereas *Candidatus* Scalindua wagneri was predominant in JLE (⁓50%; Fig. S6). In contrast, *Candidatus* Kuenenia and *Candidatus* Brocadia each comprised only an average of 1.1% of the total anammox bacteria, with the highest relative abundances found in the JLE, which also has the maximum ammonium concentrations (Fig. 2B). *Candidatus* Brocadia comprised four species: *Candidatus* Brocadia anammoxidans, *Candidatus* Brocadia caroliniensis, *Candidatus* Brocadia fulgida, and *Candidatus* Brocadia sapporoensis. *Candidatus* Kuenenia had only one species, *Candidatus* Kuenenia stuttgartiensis (Fig. S6).

### Variations in enriched anammox bacteria species across coastal sediments

**Fig. 3:**
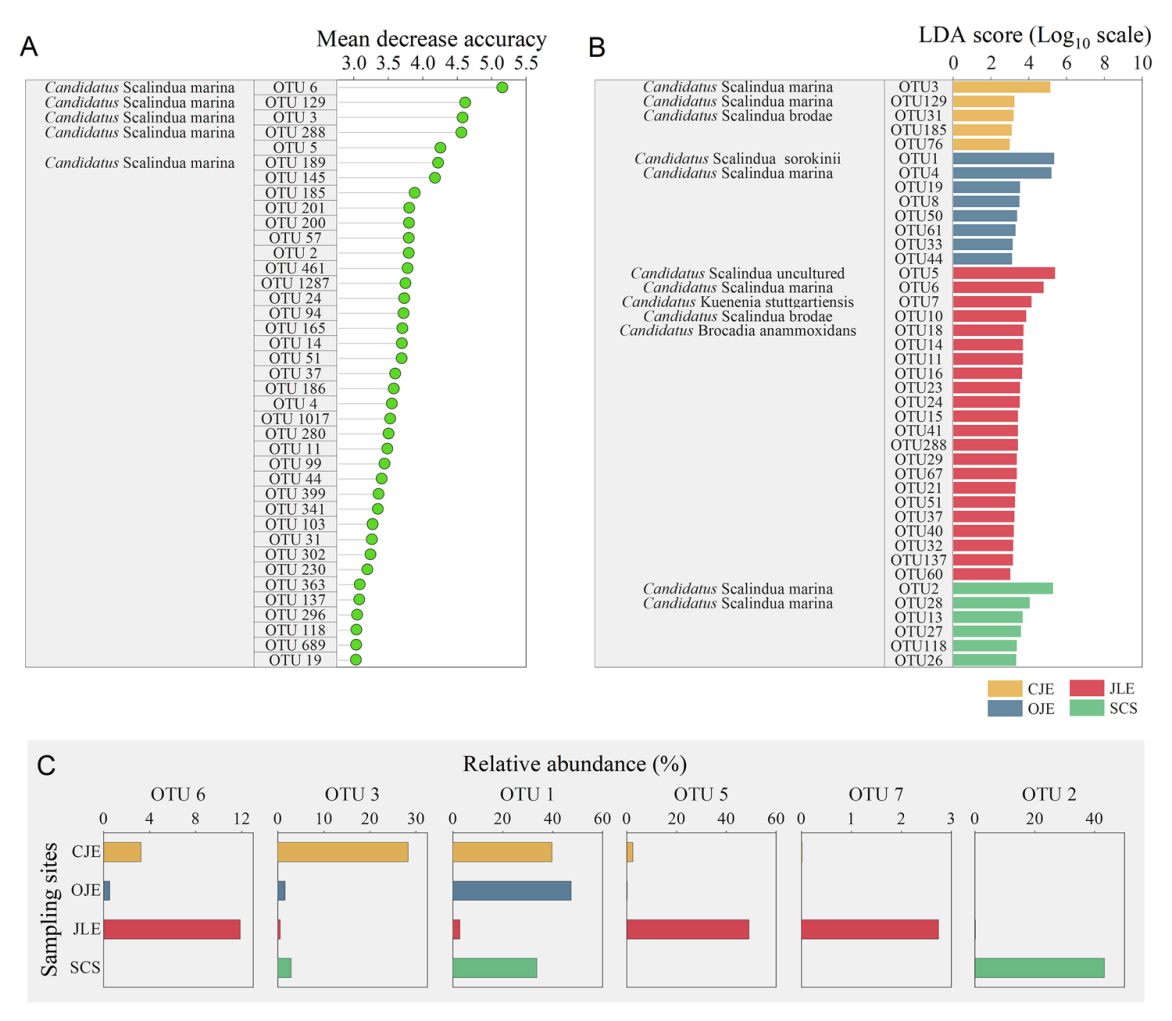
Enrichment preferences of the anammox bacteria operational taxonomic units (OTUs) among different sediments. **A** Mean decreased accuracy of the OTUs that significantly contributed to categorizing various ecosystems. **B** Indicator anammox bacteria species in different environments with linear discriminant analysis (LDA) scores higher than 2.0. **C** Relative abundances of the typical indicator anammox bacteria species (enriched OTUs) across varied ecosystems. *CJE* Changjiang Estuary, *OJE* Oujiang Estuary, *JLE* Jiulong River Estuary, *SCS* South China Sea

We identified the OTUs that were remarkably enriched in the different coastal sediments using RFA and LDA (Fig. 3). The RFA results revealed that OTU6, belonging to *Candidatus* Scalindua marina, had the greatest influence in driving community heterogeneity (Fig. 3A). LDA results indicated that OTU3, belonging to *Candidatus* Scalindua marina, was preferentially enriched in the CJE. OTU1, belonging to *Candidatus* Scalindua sorokinii, accumulated in the OJE sediments. Notably, besides the two marine species (OTU5 and 6) being the primary differentiators with the greatest LDA scores, OTU7 (*Candidatus* Kuenenia stuttgartiensis), a freshwater genus, was also a key differentiator in JLE. OTU2, from *Candidatus* Scalindua marina, was identified as a significant differential species for SCS sediments (Fig. 3B). The relative abundances of these typically enriched species, identified through RFA and LDA, across regional sediments are shown in Fig. 3C.

### Spatial distribution characteristics of the anammox bacterial communities

**Fig. 4:**
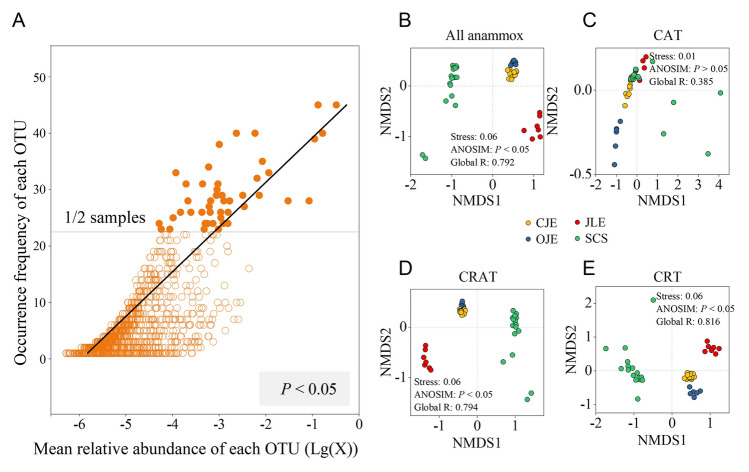
**A** Occurrence frequency of each operational taxonomic unit (OTU). **B** Nonmetric multidimensional scaling analysis of the total anammox bacterial communities, **C** conditionally abundant taxa (CAT), **D** conditionally rare or abundant taxa (CRAT), and **E** conditionally rare taxa (CRT). *CJE* Changjiang Estuary, *OJE* Oujiang Estuary, *JLE* Jiulong River Estuary, *SCS* South China Sea

Analysis of the occurrence patterns revealed that only ~ 1% (46/4363) of the OTUs were present in more than 50% of the samples (Fig. 4A). The analysis showed a significant positive relationship between the mean relative abundance and occurrence frequency of the OTUs, suggesting that abundant species were more likely to disperse, whereas rare species had a weaker ability to occupy nearby locations (Fig. 4A). Additionally, OTUs from the various ecosystems exhibited similar occurrence patterns (Fig. S7). Different community assembly mechanisms may exist between the abundant and rare taxa. Based on the Bray–Curtis dissimilarity, the NMDS and ANOSIM analyses of the large-scale spatial heterogeneity in anammox bacterial communities revealed evident spatial differences among the areas, with samples from the same area clustering (Fig. 4B; ANOSIM: *P* < 0.05). NMDS analysis of the rare species indicated that these communities displayed a greater spatial heterogeneity (higher Global R) compared to the overall anammox bacterial community. (Fig. 4C–E).

### Community assembly mechanisms of the anammox bacteria

**Fig. 5:**
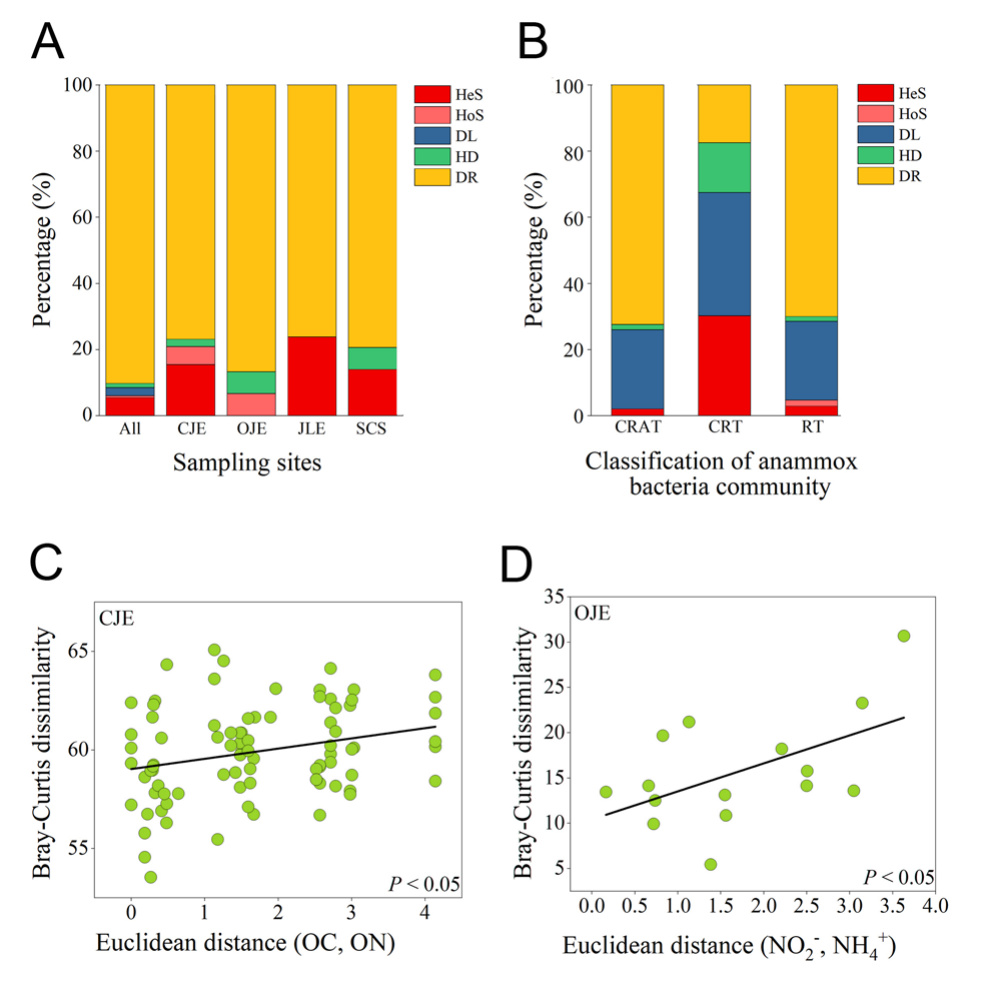
**A** Relative importance of the stochastic and deterministic processes involved in the assembly of the total anammox bacterial community. **B** Rare taxa (RT), conditionally rare taxa (CRT), and conditionally rare or abundant taxa (CRAT). **C** Correlations between the Euclidean distance of the environmental factors and Bray–Curtis dissimilarity of the anammox bacteria communities in the Changjiang Estuary (CJE) and **D** Oujiang Estuary (OJE). *JLE* Jiulong River Estuary, *SCS* South China Sea, *HeS* heterogeneous selection, *HoS* homogeneous selection, *DL* dispersal limitation, *HD* homogenizing dispersal, *DR* Drift, NO_x_^–^ nitrite + nitrate, NH_4_^+^ ammonium, *ON* organic nitrogen, *OC* organic carbon

The community assembly mechanisms of anammox bacteria were analyzed utilizing a null model, which indicated that ecological drift was the primary driver, contributing ~ 90% to the ecological processes (Fig. 5A). Heterogeneous selection played a more significant role at smaller scales (within JLE, CJE, and SCS), whereas dispersal limitation greatly impacted at larger scales (all data) (Fig. 5A). Notably, homogeneous dispersal and selection contributed to anammox bacterial community assembly in OJE, without any influence from heterogeneous processes. Rare anammox bacteria species exhibited distinct assembly mechanisms. Ecological drift and dispersal limitation primarily influenced the CRAT (72.4% and 24%, respectively) and RT (70.1% and 23.8%, respectively), whereas in the CRT, dispersal limitation (37.2%) and heterogeneous selection (30.3%) were the dominant ecological processes (Fig. 5B). In addition, we analyzed the correlation between environmental parameters and the community structure. The results revealed that organic matter (organic carbon and nitrogen) and anammox substrates (NO_2_^–^ and NH_4_^+^) markedly impacted the community heterogeneity of anammox bacteria in the CJE and OJE, respectively (Fig. 5C and D). In summary, ecological drift emerged as the dominant process driving the assembly of the anammox bacterial communities. However, rare species were relatively more influenced by dispersal limitation and heterogeneous selection. Stochastic processes, along with environmental factors, concurrently shaped the anammox bacterial community structure in the coastal sediments.

### Spatial heterogeneity of anammox bacterial co-occurrence networks

**Fig. 6:**
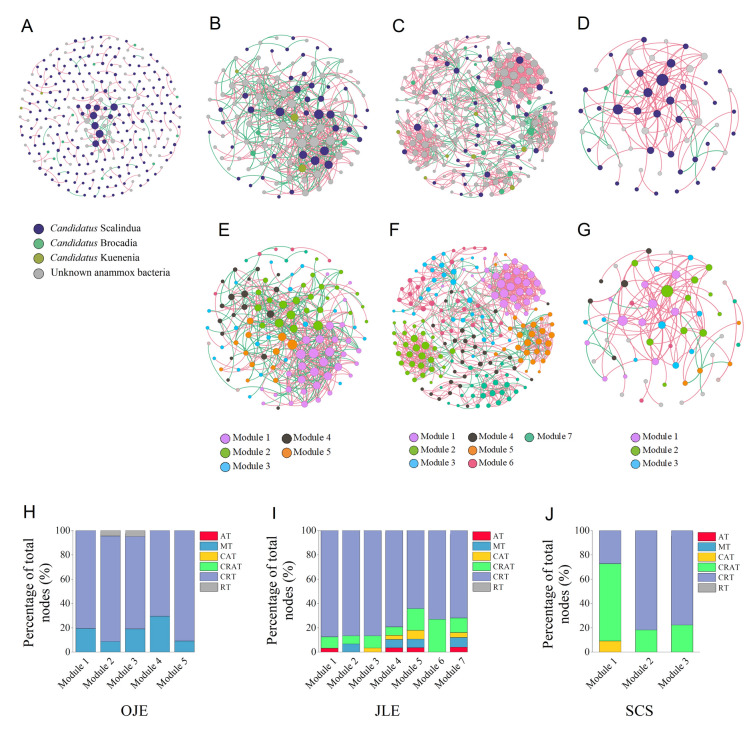
**A** Co-occurrence networks of the anammox bacterial community based on Spearman’s correlations between the operational taxonomic units (OTUs) in the Changjiang Estuary (CJE), (**B** and **E**) Oujiang Estuary (OJE), (**C** and **F**) Jiulong River Estuary (JLE), and (**D** and **G**) South China Sea (SCS). Only connections with a correlation coefficient greater than 0.6 and a *P* value lower than 0.05 are shown. Nodes represent OTUs, and the size of each node denotes the number of correlation edges. Nodes in **A**–**D** are colored by taxonomy, and nodes in **E**–**G** are colored by modularity. The red and green lines represent positive and negative correlations, respectively. **H** Proportions of abundant taxa (AT), moderate taxa (MT), conditionally abundant taxa (CAT), conditionally rare or abundant taxa (CRAT), conditionally rare taxa (CRT), and rare taxa (RT) among the total nodes within the different modules in OJE, **I** JLE, and **J** SCS

The structures and topological parameters of the anammox bacteria networks across different coastal sediments are shown in Fig. 6A–D and Table 1. Overall, OJE and JLE exhibited greater network diameters and complexity compared to CJE and SCS. The JLE network exhibited the maximum number of correlation edges (945), average path length (4.08), network diameter (9), and average degree (9.17). In contrast, the SCS had the highest betweenness centrality (0.15) and degree centrality (0.22), indicating that most nodes functioned as intermediaries or bridges between other nodes within the network. Positive correlation edges accounted for 80% of the total relationships in the CJE anammox bacteria network, 48.4% in the OJE network, 76.1% in the JLE network, and 90% in the SCS network. This result indicates a predominance of mutualism or cooperation within anammox bacterial interactions, except for OJE. Modularity analysis revealed five, seven, and three main modules in the OJE, JLE, and SCS networks, respectively, while no major modules were identified in the CJE network (Fig. 6E–G). CRT was the primary component in each module, constituting 71%–91% in OJE, 64%–88% in JLE, and 78%–82% in SCS, except for module 1 in the SCS network, where CRAT dominated (Fig. 6H–J). These results demonstrate the essential role of rare species in the anammox bacterial ecological network.

**Table 1:** Topological parameters of the co-occurrence networks of the anammox bacterial communities in different ecosystems

Topological parameters	CJE	OJE	JLE	SCS
Number of edges	202	504	945	110
Number of vertices	285	119	206	59
Average degree	1.41754	8.47059	9.17476	3.72881
Average path length	3.18453	3.11769	4.08184	2.69912
Diameter	7	8	9	7
Clustering coefficient	0.26769	0.45843	0.61771	0.43308
Betweenness centralization	0.01182	0.06516	0.13003	0.1459
Degree centralization	0.03	0.16	0.09	0.22
Modularity	0.9	0.42	0.75	0.48
Graph density	0.005	0.072	0.045	0.064
Number of positive links	161 (80%)	244 (48.4%)	719 (76.1%)	99 (90%)
Number of negative links	41 (20%)	260 (51.6%)	226 (23.9%)	11 (10%)
Proportion of Scalindua (%)	71.58	34.45	20.39	57.63
Proportion of Brocadia (%)	3.86	2.52	5.34	3.39
Proportion of Kuenenia (%)	0.7	2.52	3.4	0
Proportion of Unclassified Anammox (%)	23.86	60.5	70.87	38.98

*CJE* Changjiang Estuary, *OJE* Oujiang Estuary, *JLE* Jiulong River Estuary, *SCS* South China Sea

**Fig. 7:**
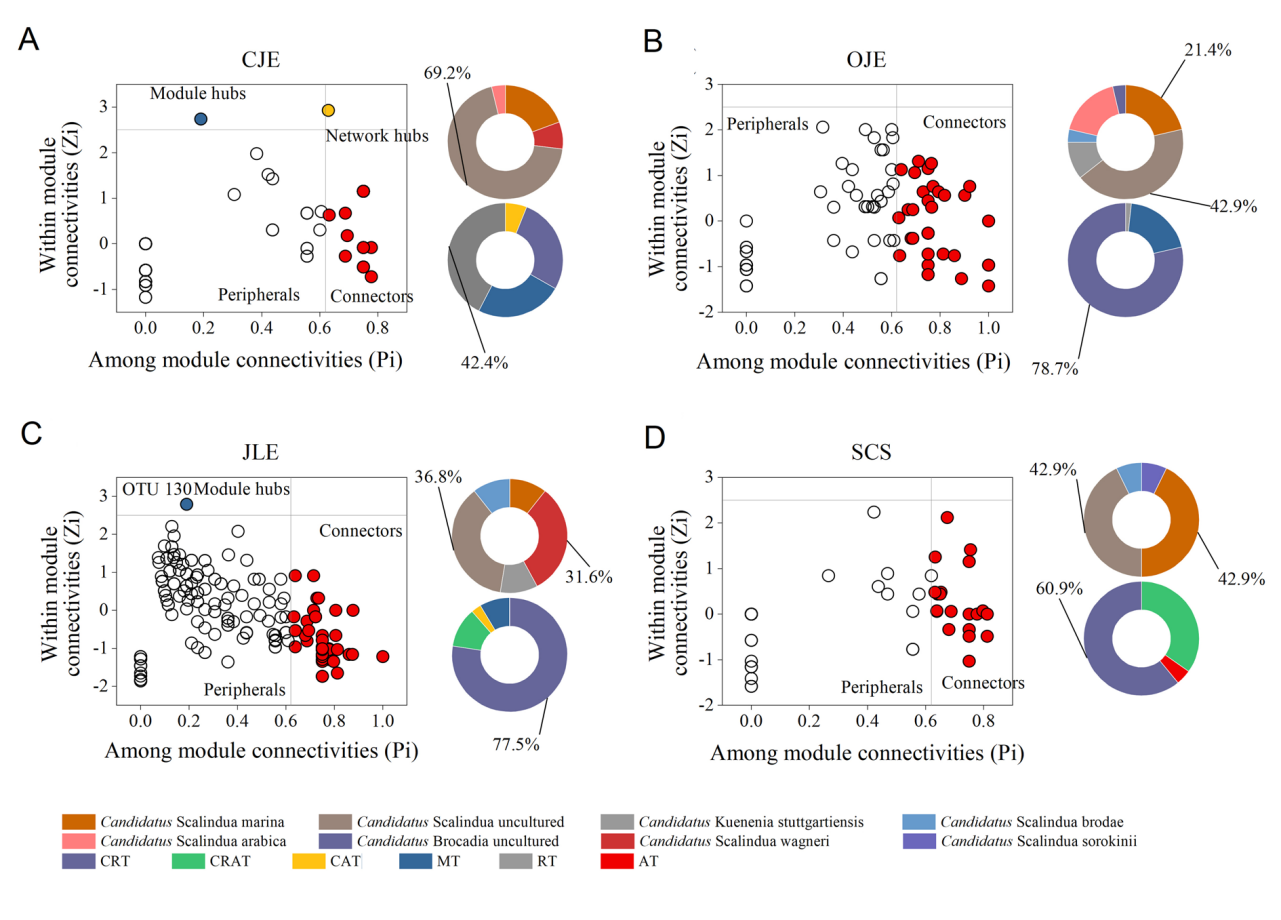
**A** Pi-Zi plots of the anammox bacterial community network in the Changjiang Estuary (CJE), **B** Oujiang Estuary (OJE), **C** Jiulong River Estuary (JLE), and **D** South China Sea (SCS). The taxonomy (color legend) and composition proportions of the connector operational taxonomic units are displayed on the right side of the Pi-Zi plots as pie charts. *AT* abundant taxa, *MT* moderate taxa, *CAT* conditionally abundant taxa, *CRAT* conditionally rare or abundant taxa, *CRT* conditionally rare taxa, *RT* rare taxa

The Pi-Zi plot illustrated the keystone species in different ecosystems (Fig. 7). The CJE displayed 26 connectors, predominantly involving unknown *Candidatus* Scalindua spp. (69.2%). An unidentified species from *Candidatus* Scalindua was recognized as a module hub, while an OTU belonging to *Candidatus* Scalindua sorokinii served as a network hub, exemplifying the primary role of *Candidatus* Scalindua sorokinii in CJE (Fig. 7A). Additionally, 28 connectors were observed in OJE, consisting primarily of *Candidatus* Scalindua uncultured (42.9%), *Candidatus* Scalindua marina (21.4%), and *Candidatus* Scalindua arabica (17.9%) (Fig. 7B). In JLE, one network hub (OTU130, *Candidatus* Brocadia) and 19 connectors were identified. Of these, 36.8% were *Candidatus* Scalindua uncultured, and 31.6% were *Candidatus* Scalindua wagneri (Fig. 7C). In SCS, 14 connectors were identified, with unknown *Candidatus* Scalindua (42.9%) and *Candidatus* Scalindua marina (42.9%) (Fig. 7D). Notably, CRT was the predominant keystone, accounting for 78.7%, 77.5%, and 60.9% of the total connectors in the anammox bacterial networks of OJE, JLE, and SCS, respectively (Fig. 7A–D). Furthermore, RT accounted for 42.4% of the CJE network connectors. These findings emphasize the critical role of rare species in the anammox bacterial ecological network within coastal sediments.

## Discussion

### Highly diverse anammox bacteria in estuarine environments

Estuarine ecosystems exhibited significantly higher species diversity of anammox bacteria compared with marine ecosystems (ANOVA, *P* < 0.05) (Fig. S8). The alpha diversity of anammox bacteria in the sediments of the Jiaozhou Bay, Pearl River Estuary, and CJE was greater than that in the sediments of Chinese marginal seas and the Eastern Indian Ocean (Dang et al. 2010; Fu et al. 2019; Qian et al. 2018; Shehzad et al. 2016; Wu et al. 2019a). Such high alpha diversity in estuarine environments is due to their unique ecological and hydrological characteristics, which facilitate the mixing of marine and terrestrial anammox bacteria through river runoff and tidal transport, thus enhancing species diversity and richness (Hou et al. 2013). Human activities and livestock farming near estuaries often lead to eutrophication, which supports more diverse anammox bacterial communities in these environments (Naeher et al. 2015; Wu et al. 2019a). In this study, the NH_4_^+^ concentration in JLE was much higher than that in many other sediments reported previously (Jiao et al. 2018; Russ et al. 2013; Wu et al. 2019b). JLE displayed the highest Shannon diversity and NTI indices among all the samples. Furthermore, network stability analysis confirmed the maximal ecological stability in JLE and the lowest in SCS, indicating that wider alpha diversity shaped greater network complexity and ecological stability (Fig. S9).

### Versatile ecological adaptability of the anammox bacteria

*Candidatus* Scalindua is the dominant genus in coastal sediments, exhibiting the highest relative abundance in SCS. A substantial body of research has confirmed that it is prevalent in marine environments (Dang et al. 2013; Hirsch et al. 2011). For example, an analysis of 170 global ocean oxygen minimum zone (OMZ) samples revealed the exclusive presence of *Candidatus* Scalindua in marine OMZs (Woebken et al. 2008). They are believed to potentially adapt to the ocean’s minimal nutrient levels, low temperatures, high salinity, and enrichment of refractory organic matter (Jaeschke et al. 2009; Russ et al. 2013; van de Vossenberg et al. 2008). Notably, the initial species of *Candidatus* Scalindua, namely *Candidatus* Scalindua brodae and *Candidatus* Scalindua wagneri, were enriched and isolated using density gradient centrifugation from bioreactors rather than marine ecosystems. These findings suggest the versatile metabolic capability and ecological adaptability of *Candidatus* Scalindua across diverse environments (Schmid et al. 2003).

The ecological adaptability of *Candidatus* Scalindua is facilitated by unique molecular mechanisms, such as the enhanced expression of high-affinity NH_4_^+^ transporters, formate/NO_2_^–^ transporters, and genes involved in oligopeptide and amino acid transport, enabling its widespread distribution in low-nutrient environments (van de Vossenberg et al. 2013). *Candidatus* Scalindua exhibits diverse metabolic pathways; in addition to NO_2_^–^, it can use metal oxides as electron acceptors, enabling its survival in NO_x_^–^-depleted marine environments and deep sediments (van de Vossenberg et al. 2008; van de Vossenberg et al. 2013). In environments with low levels of organic acids like propionate, acetate, formate, etc., *Candidatus* Scalindua can reduce NO_2_^–^ to NH_4_^+^ through the dissimilatory nitrate reduction to ammonium process, thus providing substrates for anammox metabolism (Lam and Kuypers 2011; van de Vossenberg et al. 2013).

**Fig. 8:**
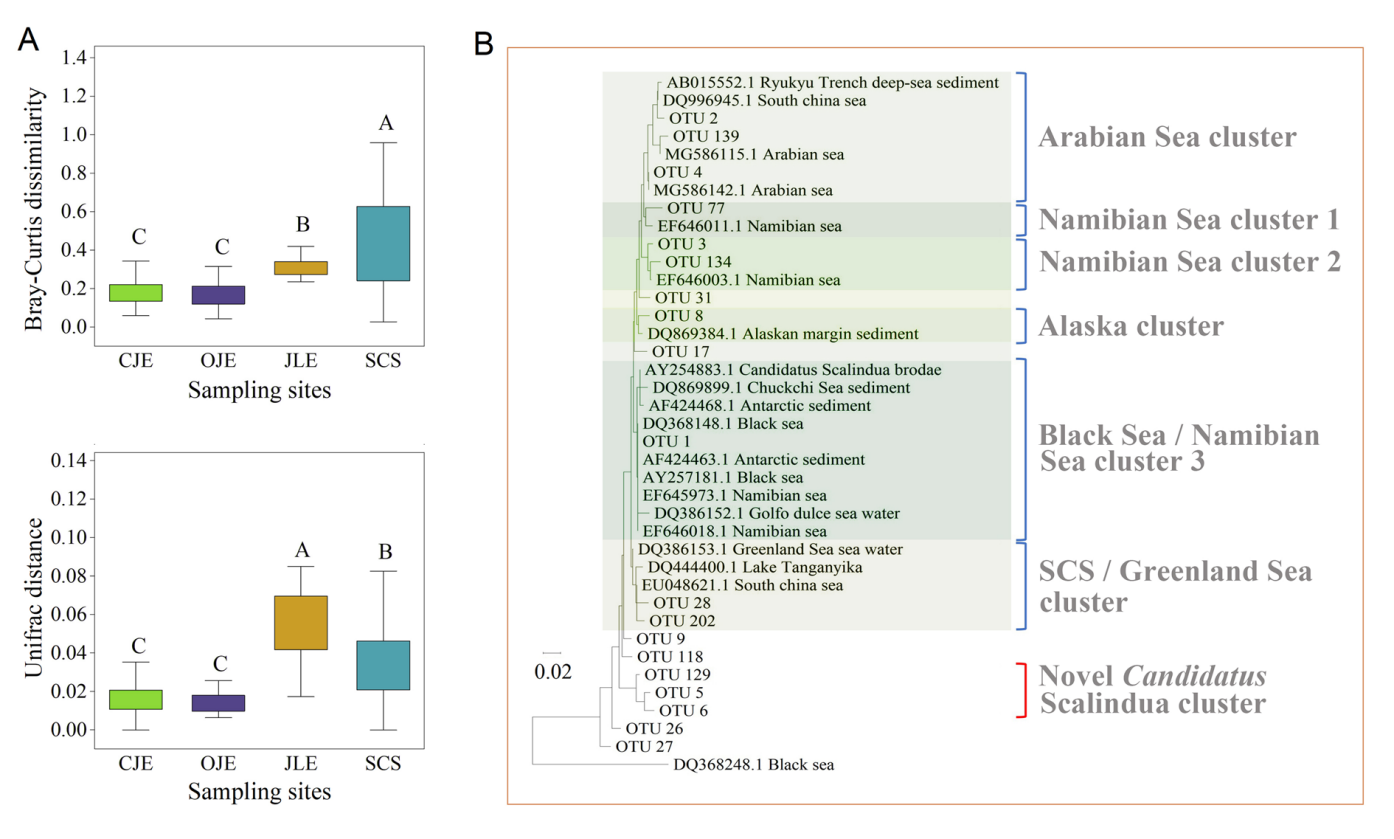
**A** Beta diversity of the anammox bacterial communities in different regions, assessed using Bray–Curtis dissimilarity and UniFrac distance. The different letters denote significant differences. **B** The phylogenetic tree of *Candidatus* Scalindua is based on the operational taxonomic units from this study and public databases. *CJE* Changjiang Estuary, *OJE* Oujiang Estuary, *JLE* Jiulong River Estuary, *SCS* South China Sea

Phylogenetics revealed that *Candidatus* Scalindua exhibited remarkably high diversity (Fig. 2A). The alpha diversity of anammox bacteria was significantly low in SCS. However, the species richness index did not differ markedly from that of other estuarine environments, except for CJE (Fig. S5). Notably, the beta diversity of the anammox bacterial communities was remarkably greater in SCS compared to other estuarine sediments, despite the UniFrac distance in JLE being higher than in SCS (Fig. 8A). These findings align with those of previous studies which indicate that a decline in alpha diversity typically broadens the beta diversity (Chase and Myers 2011; Koleff et al. 2003), suggesting significant microevolution within *Candidatus* Scalindua. To demonstrate this diversity, a phylogenetic tree of *Candidatus* Scalindua was constructed based on sequences derived from this study and those of various marine regions obtained from the NCBI (Fig. 8B). The results indicated that the sequences from this study had a high homology to those from the Arabian Sea, Namibian Sea, Alaskan waters, Black Sea, Greenland Sea, and SCS. Furthermore, potential new clades of *Candidatus* Scalindua were identified. These findings demonstrate the worldwide ecological distribution and wide diversity of *Candidatus* Scalindua.

*Candidatus* Brocadia and *Candidatus* Kuenenia exhibit a preference for distribution in terrestrial or freshwater environments due to their sensitivity to salinity and preference for high-nutrient conditions (Dale et al. 2009; Wu et al. 2019a). In this study, the relative abundance of *Candidatus* Brocadia and *Candidatus* Kuenenia in the JLE was markedly higher than those in other sediment environments (Fig. 2B), along with a greater diversity of anammox bacteria in JLE (Fig. S5). The most abundant *Candidatus* Brocadia species were *Candidatus* Brocadia anammoxidans and *Candidatus* Brocadia fulgida. *Candidatus* Brocadia fulgida can efficiently utilize acetate, showing a higher rate of organic acid utilization in co-culture systems with denitrifying bacteria and constituting more than 70% of the total community (Kartal et al. 2008). This finding suggests that *Candidatus* Brocadia can stably survive in organic compound-rich environments such as estuaries. In addition, we observed *Candidatus* Brocadia to be ~ 0.9% of the total community in the SCS. The presence of this genus in marine environments may be allochthonous, having immigrated to the SCS via river runoff and surviving, as they have a high adaptability to saline environments (Li et al. 2018).

Genome analysis of *Candidatus* Kuenenia revealed that it possesses at least three genes related to the direct conversion of organic acids to acetyl-CoA and has a high affinity for NH_4_^+^ and NO_2_^–^, enabling it to survive in various environments (Kartal et al. 2008; Strous et al. 1999, 2006).

### Deterministic and stochastic processes together shape the anammox bacterial community

Null model analysis revealed that ecological drift predominantly governed the community assembly of anammox bacteria within coastal sediments. Conversely, heterogeneous selection played a minor role in these regions, except for OJE, which was impacted by minor homogeneous effects. Heterogeneous selection and ecological drift are relative processes. Communities with robust environmental selection exhibit weakened ecological drift (Chase 2010; Chase and Myers 2011). Conversely, communities characterized by dominant ecological drift typically display weak environmental selection, meaning that they have greater ecological equivalence. It is believed that an individual NTI index less than 2 or a mean NTI index significantly greater than 0 suggests weak environmental selection, i.e., the community assembly process is dominated by stochastic processes (Stegen et al. 2012). In this study, the mean NTI values were significantly greater than 0 across all regions, and the NTI indices of the individual community were all less than 2 (Fig. S5C). These results suggested that the anammox bacteria experienced minimal environmental selection pressure and were predominantly influenced by stochastic processes, which is consistent with the null model analysis results. These findings also align with those of previous studies suggesting that ecological drift is more likely to influence smaller microbial communities, such as the anammox bacterial community, in natural environments (Chase and Myers 2011; Nemergut et al. 2013).

Notably, although heterogeneous selection played a minor role in the anammox bacterial communities based on the null model, the significant influence of environmental parameters on the community structure was demonstrated (Fig. 5C and D). Consistent with previous studies (Chen et al. 2021; Li et al. 2018), our results indicate that substrate availability (NO_2_^–^ and NH_4_^+^) plays a pivotal role in shaping the anammox bacterial community structure. Their elevated concentrations typically favor low-affinity groups, such as *Candidatus* Brocadia in JLE, whereas oligotrophic conditions favor high-affinity groups, such as *Candidatus* Scalindua in SCS (Zhang and Okabe 2020). Organic matter can also influence anammox communities through dual mechanisms: directly, by serving as a NO_2_^–^ and NH_4_^+^ source through remineralization (Hou et al. 2013; Shao et al. 2014), and indirectly, by modulating associated microbial communities, such as denitrifying bacteria (Meyer et al. 2005). Therefore, we emphasize that both stochastic processes and environmental selection jointly shape the community structure of anammox bacteria.

### Drift, dispersal limitation, and heterogeneous selection dominate the assembly of the rare anammox bacteria

Anammox bacteria displayed distinct community assembly mechanisms depending on their relative abundances. We observed that the abundant taxa exhibited robust dispersal abilities, whereas low-abundance taxa showed notable dispersal limitation, consistent with previous findings on the total bacterial community (Galand et al. 2009; Jia et al. 2018). Notably, low-abundance taxa have smaller population sizes, which inherently limits the number of individuals available to disperse to new locations (Lindström and Langenheder 2012). Furthermore, such taxa often occupy specialized or narrow ecological niches, making them less adaptable to diverse environments (Jousset et al. 2017). Consequently, the contribution of dispersal limitation to rare anammox bacteria is amplified.

Upon classifying low-abundance anammox bacteria into RT, CRT, and CRAT, we found that ecological drift dominated the community assembly processes in RT and CRAT, with dispersal limitation playing a secondary role. However, in CRT, dispersal limitation was the primary factor. These findings are consistent with previous studies suggesting that low-abundance taxa are more susceptible to ecological drift and have a limited ability to disperse (Ai et al. 2013; Hanson et al. 2012; Mo et al. 2018; Nemergut et al. 2013). Additionally, heterogeneous selection also contributed heavily to the CRT assembly. The rarity of CRT may be attributed to their sensitivity to environmental fluctuations (Gobet et al. 2012). A majority of CRT can sustain low population densities and transit into a dormant state under adverse environmental conditions (Aanderud et al. 2015). This explains the influence of heterogeneous selection on the community assembly processes. Evidently, rare anammox bacteria, which are often overlooked, play a critical role in understanding the assembly mechanisms of the entire microbial community (Allan et al. 2011; Nemergut et al. 2011).

### Significance of rare anammox bacteria within ecological networks

The keystone species within the ecological networks of anammox bacteria, primarily CRT or RT, along with the major groups forming the modules predominantly composed of CRT, underscore the significant role of rare species in these ecological networks and unique environments, suggesting their potential specialized functions (Abdullah Al et al. 2022; Banerjee et al. 2018; Li et al. 2019; Xue et al. 2018). As crucial functional microorganisms, anammox bacteria play a remarkable role in the global N budget. Although rare taxa have high functional redundancy, they may become particularly critical under altering environmental conditions, such as climate change (Fetzer et al. 2015; Rousk et al. 2009). A previous study also highlighted that denitrification relies on rare species. A 75% reduction in the species richness of samples led to a 4–fivefold decrease in the soil denitrification activity, indicating that a single abundant species is inadequate to maintain denitrification (Philippot et al. 2013). A study conducted in the Delaware coastal sea revealed that at least half of the rare taxa are active, playing an essential role in biogeochemistry (Campbell et al. 2011). Therefore, rare species, representing a large gene pool with robust metabolic potential, are essential for maintaining the stability of microbial ecological networks and functions (Jia et al. 2018; Liang et al. 2020; van Elsas et al. 2012).

Notably, periodic transitions between rare and abundant states can occur depending on changes in the environmental conditions (Lauro et al. 2009; Yooseph et al. 2010). The community structure of anammox bacteria varies with changes in salinity or organic matter, implying that their population density is influenced by external environmental conditions (Fu et al. 2019; Kartal et al. 2007b; Zheng et al. 2016). This study ascertained that *Candidatus* Scalindua was the dominant anammox bacteria in coastal sediments, while the two freshwater genera, *Candidatus* Brocadia and *Candidatus* Kuenenia, had relatively low abundances. However, the latter are never consistently low in abundance over any time series; their relative abundances may undergo periodic changes due to human activity and environmental fluctuations. For example, varying levels of human activity can greatly impact the ecological stoichiometry of microbial N uptake, resulting in varied nutrient limitations for nitrogen-cycling microorganisms (Stelzer and Scott 2018; Wells et al. 2018). In this study, the relative abundance of *Candidatus* Kuenenia was significantly higher in JLE with the maximum ammonium concentration among all sediment environments. Alterations in nutrient conditions may result in fluctuations in the relative abundance of *Candidatus* Kuenenia and periodic transformations in the anammox bacterial community structure.

## Conclusions

This study elucidated distinct patterns of the anammox bacterial diversity, community structure, and spatial heterogeneity in three estuaries along the Chinese coastline — the CJE, OJE, and JLE — as well as in the SCS, emphasizing the ecological processes of community assembly, co-occurrence networks, and the roles of rare species. Our findings revealed that the estuarine sediments exhibited broad species diversity of anammox bacteria, while the SCS sediments displayed greater species. The versatile metabolic capabilities and ecological adaptability of *Candidatus* Scalindua have established it as the dominant anammox bacteria genus within these sediments, ranging from estuaries to the SCS, highlighting its diverse phylogenetic microevolution. Ecological drift dominated the anammox bacterial community assembly. Moreover, rare species were more susceptible to dispersal limitation and environmental selection, playing a keystone role in the co-occurrence networks of anammox bacteria. This study revealed the critical role of often overlooked rare species of anammox bacteria in upholding ecosystem stability and functionality, thereby advancing our understanding of anammox bacteria community assembly mechanisms and community dynamics in adapting to environmental changes. 

## Supplementary Information

Below is the link to the electronic supplementary material.

Supplementary file1 (DOCX 10148 KB)

## Data Availability

The raw sequence reads from this study have been deposited in the NCBI Sequence Read Archive (SRA) database under the BioProject accession number PRJNA1142181. The corresponding BioSample accessions range from SRR30046140 to SRR30046184 (https://www.ncbi.nlm.nih.gov/sra/PRJNA1142181).
