# Supplementary material for: Critical roles of rare species in the anaerobic ammonium oxidizing bacterial community in coastal sediments
Source: Mar Life Sci Technol. 2025 Jul 17;7(3):507–22. doi: 10.1007/s42995-025-00315-8 (PMC12413392; doi:10.1007/s42995-025-00315-8)
Supplement: Supplementary file 1 — Supplementary file1 (DOCX 10148 KB) [file 42995_2025_315_MOESM1_ESM.docx]

**Critical roles of rare species in anaerobic ammonium oxidation (anammox) bacterial community in coastal sediments**

Marine Life Science & Technology (MLST)

Yu Zhang^1^, Mingming Chen^1^, Rui Du^1^, Ehui Tan^2^, Shuh-Ji Kao^1,2^, Yao Zhang^*1^

^1^State Key Laboratory of Marine Environmental Science and College of Ocean and Earth Sciences, Xiamen University, Xiamen 361005, China

^2^State Key Laboratory of Marine Resource Utilization in South China Sea, School of Marine and Engineering, Hainan University, Haikou, Hainan, 570228, China

^*^Corresponding author. E-mail: yaozhang@xmu.edu.cn.


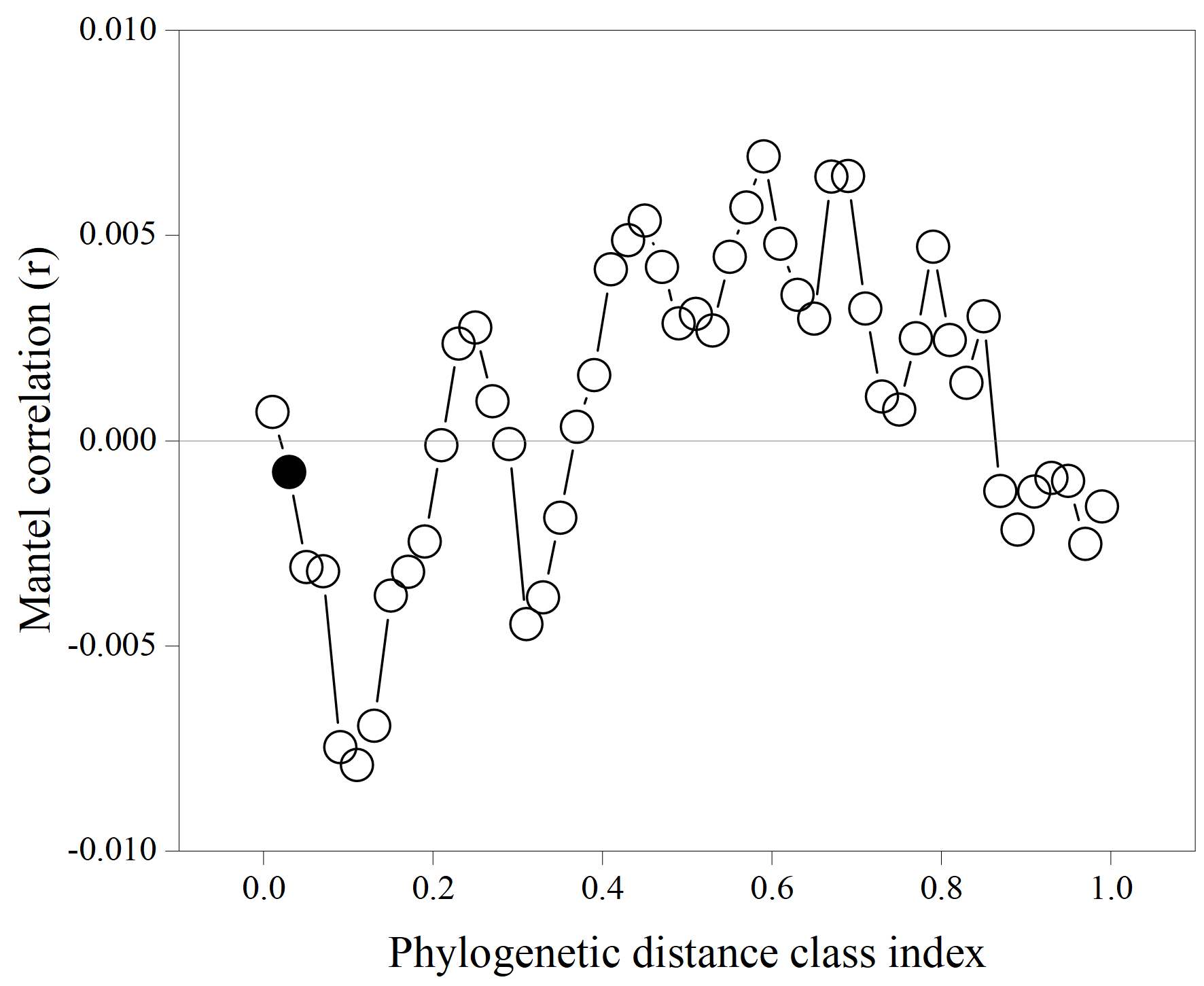


**Supplementary Fig. S1** Phylogenetic mantel correlogram showing a significant phylogenetic signal in a close phylogenetic distance. Solid and open circles represent significant and nonsignificant signals, respectively


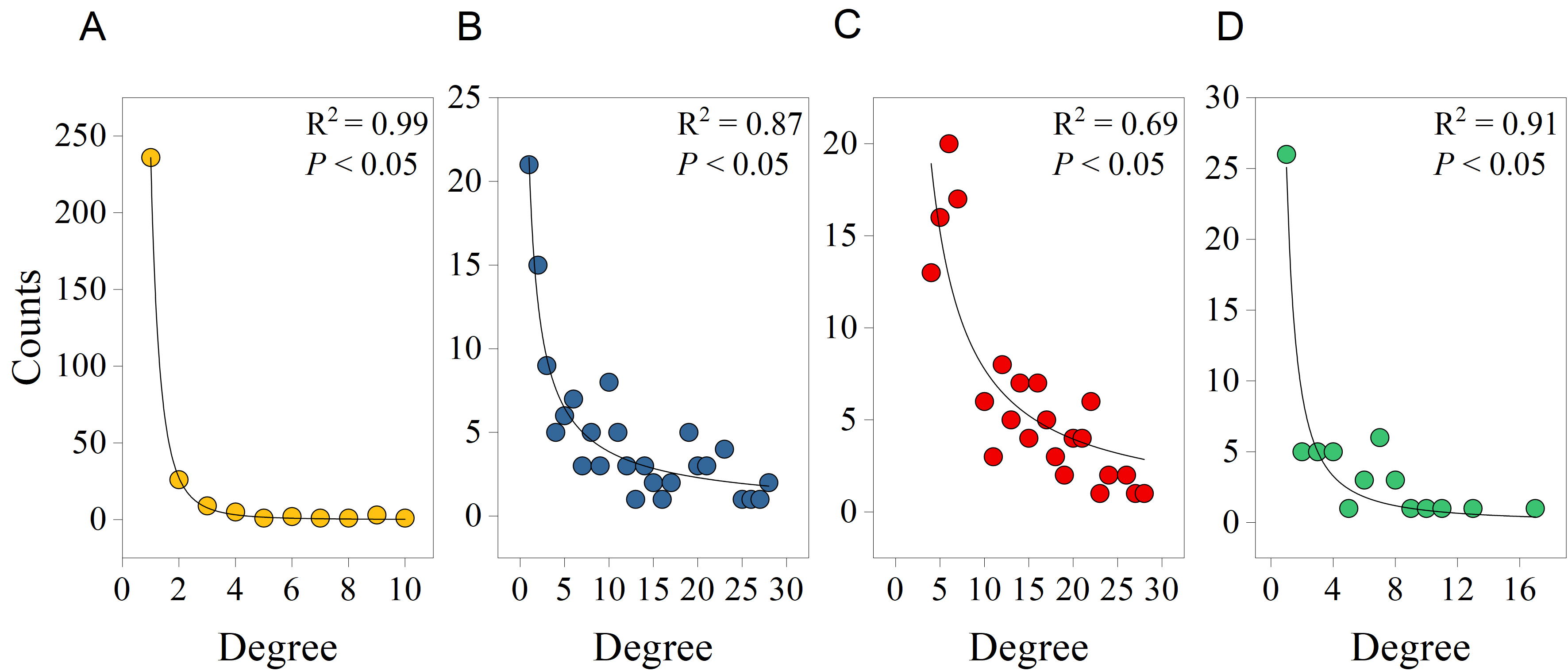


**Supplementary Fig. S2** (A) The frequency of nodes (‘Counts’) as a function of their degree, fitted with power-law curves, in the Changjiang Estuary, (B) Oujiang Estuary, (C) Jiulong River Estuary, and (D) South China Sea co-occurrence network models


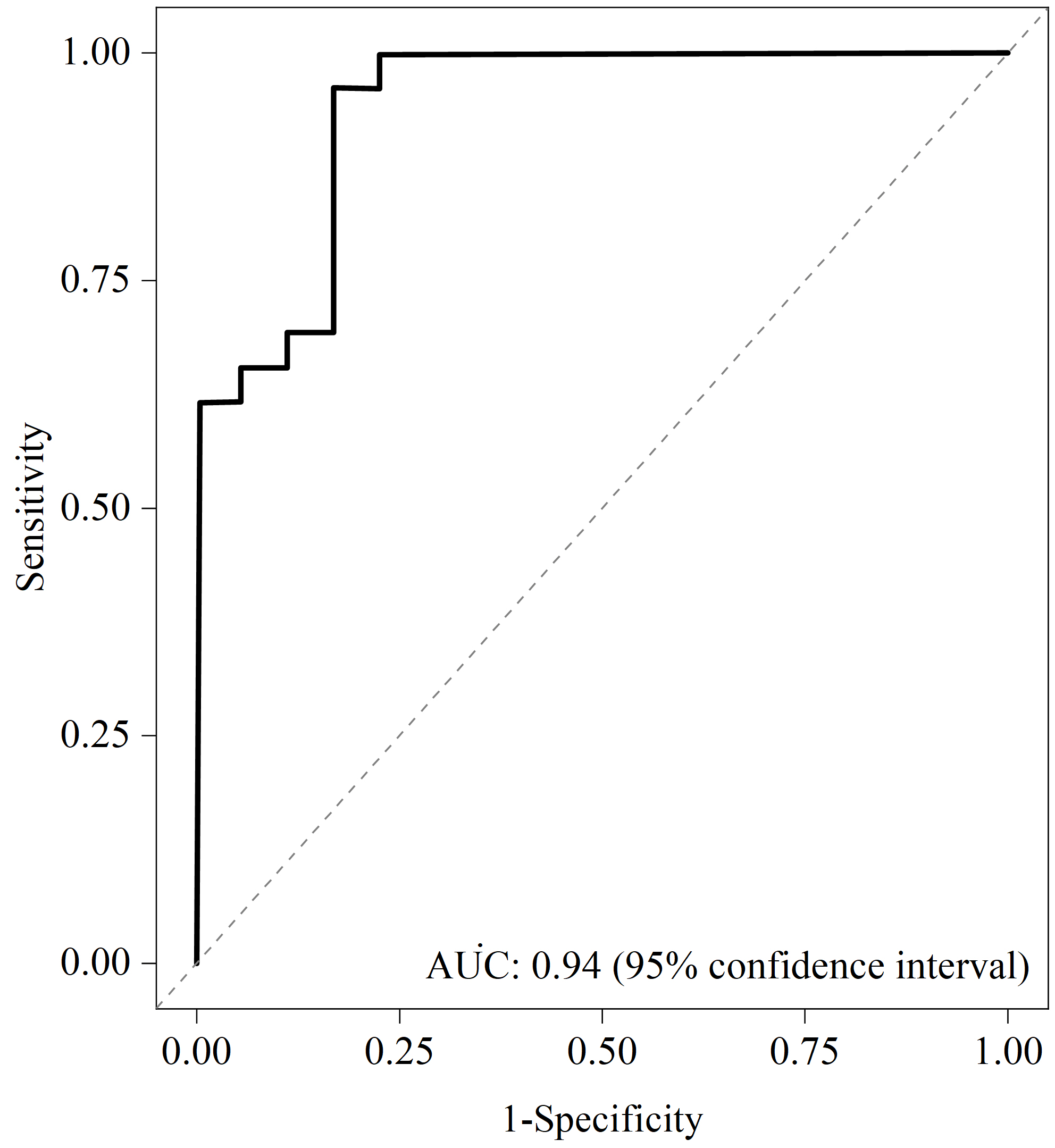


**Supplementary Fig. S3** Receiver operating characteristic curves via random forest analysis. The X-axis and Y-axis denote the false positive rate and true positive rate, respectively. The area under the curve (AUC) is 0.94, indicating a high level of diagnostic accuracy. The dashed line represents the baseline for the prediction of random forest analysis, with a AUC value of 0.5, indicating that the model’s performance is no better than chance

**
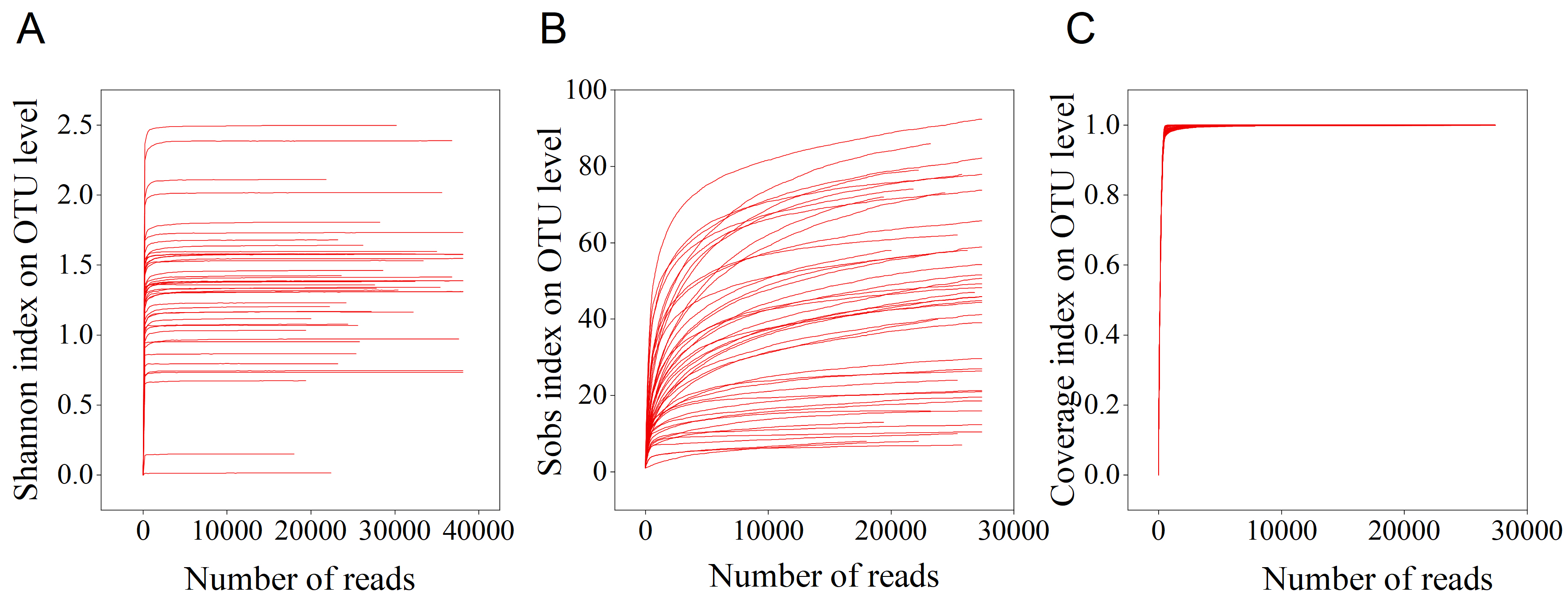
**

**Supplementary Fig. S4** (A) Rarefaction curves based on Shannon index, (B) Sobs index, and (C) Coverage index of OTUs

**
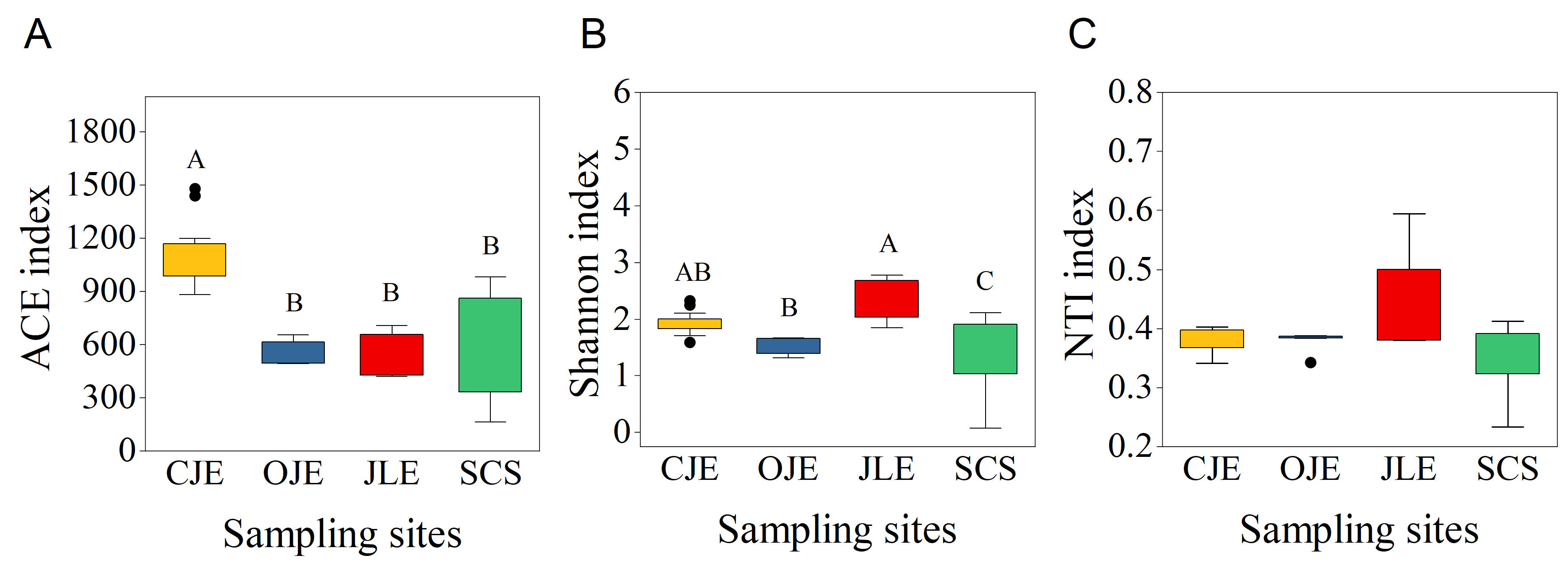
**

**Supplementary Fig. S5** Alpha diversity [(A) Shannon, (B) ACE, and (C) NTI indices] of anammox bacterial communities in the Changjiang Estuary (CJE), Oujiang Estuary (OJE), Jiulong River Estuary (JLE), and South China Sea (SCS). Different letters above the boxes denote statistically significant differences between the sampling regions


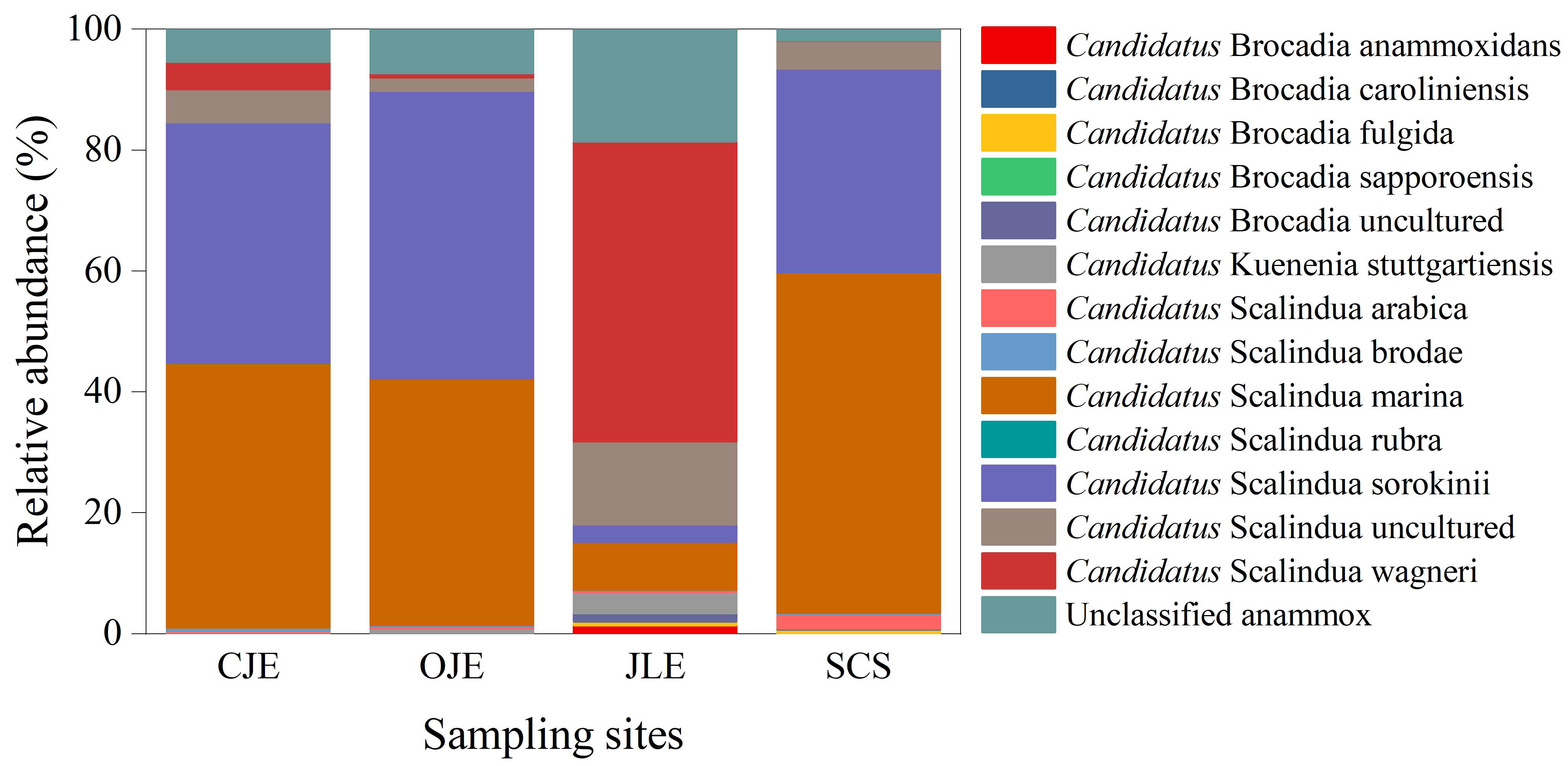


**Supplementary Fig. S6** Relative abundance of anammox bacterial species across the four sampling regions. CJE, Changjiang Estuary; OJE, Oujiang Estuary; JLE, Jiulong River Estuary; SCS, South China Sea


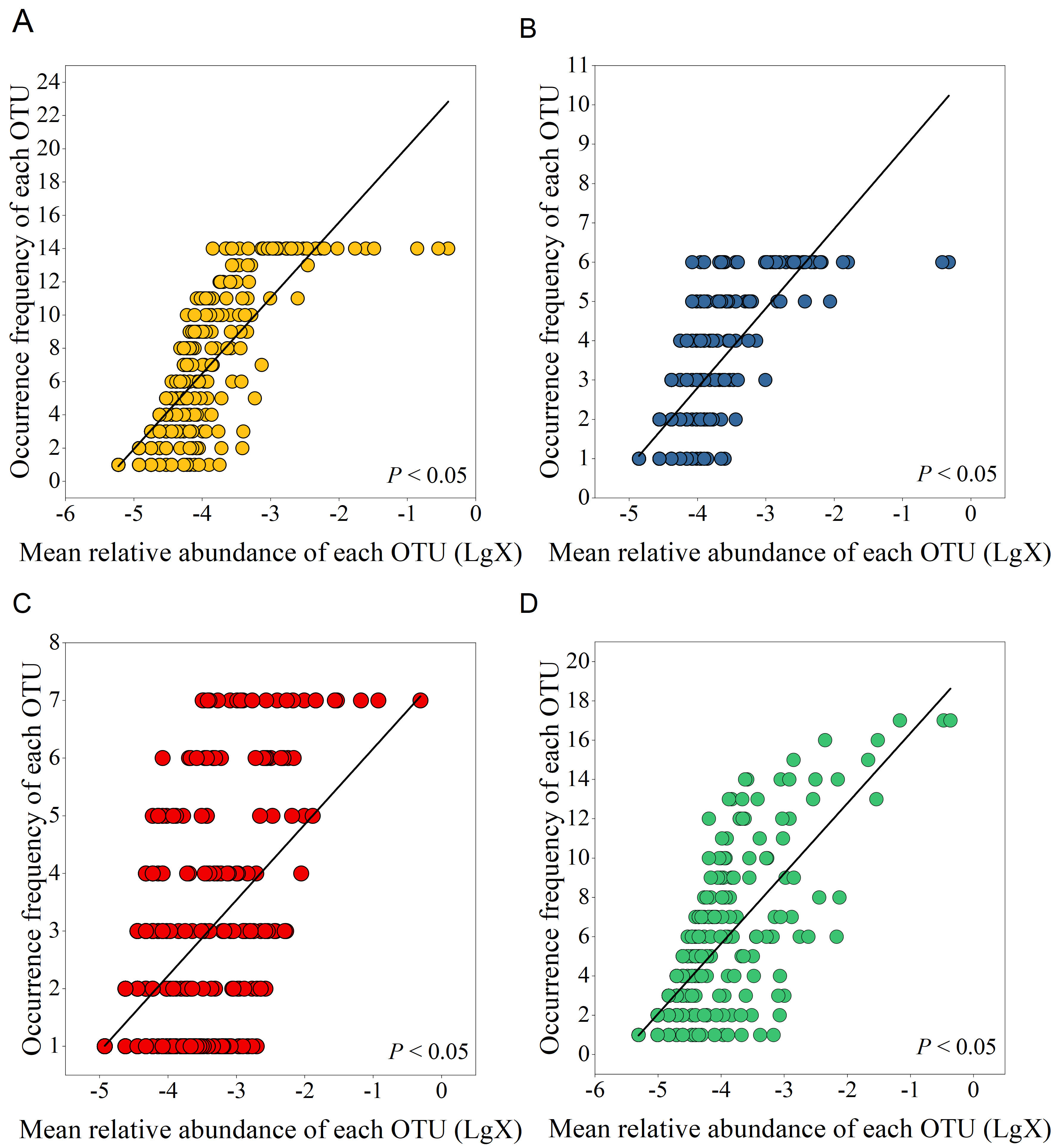


**Supplementary Fig. S7** (A) Regression analysis between occurrence frequency versus mean relative abundance of each OTU in the Changjiang Estuary, (B) Oujiang Estuary, (C) Jiulong River Estuary, and (D) South China Sea


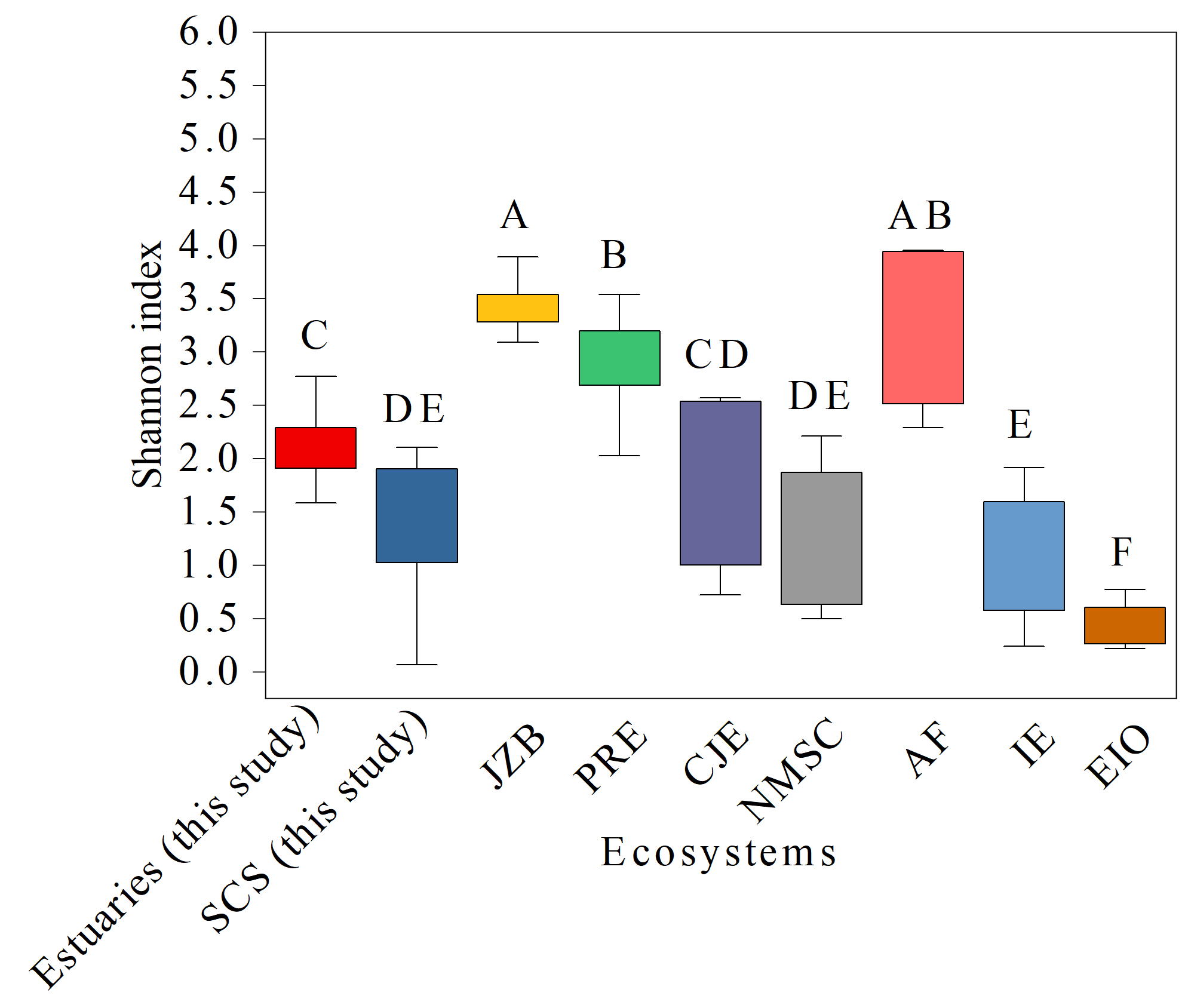


**Supplementary Fig. S8** Shannon index of anammox bacterial communities in this study and other ecosystems from the literature. Different letters above the boxes denote statistically significant differences between the estuaries and South China Sea. JZB, Jiaozhou Bay; PRE, Pearl River Estuary; CJE, Changjiang Estuary; NMSC, north marginal seas of China; AF, Arctic fjord (Vipindas et al. 2020); IE, Indus estuary (Fozia et al. 2020); EIO, eastern Indian Ocean

**
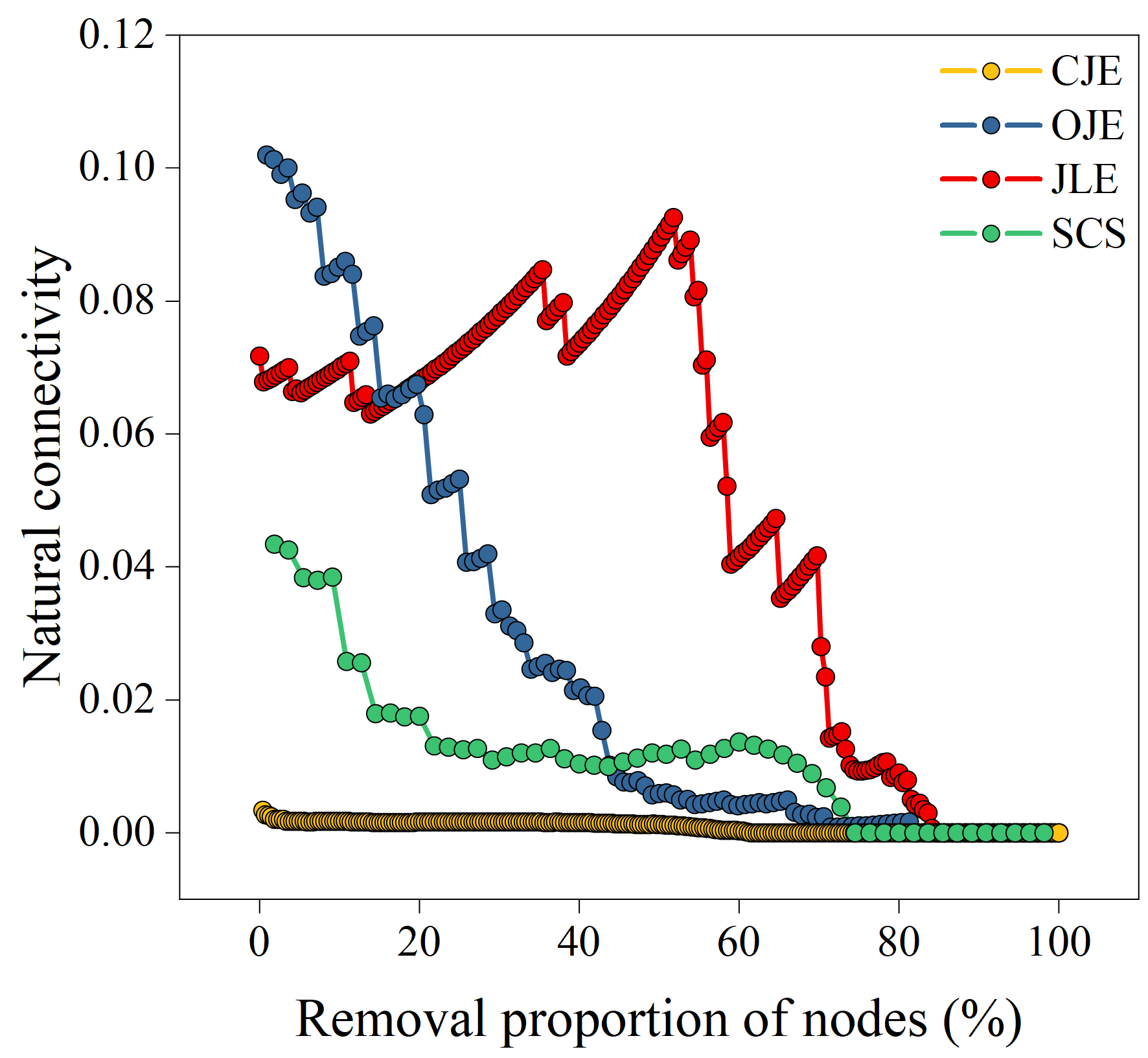
**

**Supplementary Fig. S9** Network stability analysis based on topological structure. The X-axis denotes the removal proportion of nodes, and the Y-axis denotes the natural connectivity after the nodes are removed. The curves depict how the connectivity of each network decreases with an increasing percentage of nodes removed. CJE, Changjiang Estuary; OJE, Oujiang Estuary; JLE, Jiulong River Estuary; SCS, South China Sea
